# The recombinant pseudorabies virus expressing African swine fever virus CD2v protein is safe and effective in mice

**DOI:** 10.1186/s12985-020-01450-7

**Published:** 2020-11-16

**Authors:** Zhihua Feng, Jianghua Chen, Wangwang Liang, Wenzhi Chen, Zhaolong Li, Qi Chen, Shaoli Cai

**Affiliations:** 1 Fujian Key Laboratory of Innate Immune Biology, Biomedical Research Center of South China, Fujian Province, Fujian Normal University Qishan Campus, College Town, Fuzhou, 350117 People’s Republic of China; 2 Institute of Animal Husbandry and Veterinary Medicine, Fujian Province, Fujian Academy of Agricultural Sciences, Pudang, Jin-an District, Fuzhou, 350117 People’s Republic of China

**Keywords:** African swine fever virus, CD2v, Pseudorabies virus, vaccine, immunity

## Abstract

**Background:**

African swine fever (ASF) leads to high mortality in domestic pigs and wild boar and is caused by the African swine fever virus (ASFV). Currently, no vaccine is commercially available for prevention, and the epidemic is still spreading. Here, we constructed a recombinant pseudorabies virus (PRV) (PRV-ΔgE/ΔgI/ΔTK-(CD2v)) that expresses the CD2v protein of ASFV and evaluated its effectiveness and safety as a vaccine candidate in mice.

**Methods:**

A homologous recombination fragment containing ASFV CD2v was synthesized and co-transfected into HEK 293 T cells, a knockout vector targeting the PRV TK gene. The transfected cells were infected with PRV-ΔgE/ΔgI, and the recombinant strain (PRV-ΔgE/ΔgI/ΔTK-(CD2v)) was obtained by plaque purification in Vero cells. The expression of ASFV CD2v in the recombinant virus was confirmed by sequencing, Western blotting, and immunofluorescence analysis, and the genetic stability was tested in Vero cells over 20 passages. The virulence, immunogenicity and protective ability of the recombinant virus were further tested in a mouse model.

**Results:**

The PRV-ΔgE/ΔgI/ΔTK-(CD2v) recombinant strain is stable in Vero cells, and the processing of CD2v does not depend on ASFV infection. The vaccination of PRV-ΔgE/ΔgI/ΔTK-(CD2v) causes neither pruritus, not a systemic infection and inflammation (with the high expression of interleukin-6 (IL6)). Besides, the virus vaccination can produce anti-CD2v specific antibody and activate a specific cellular immune response, and 100% protect mice from the challenge of the virulent strain (PRV-Fa). The detoxification occurs much earlier upon the recombinant virus vaccination and the amount of detoxification is much lower as well.

**Conclusions:**

The PRV-ΔgE/ΔgI/ΔTK-(CD2v) recombinant strain has strong immunogenicity, is safe and effective, and maybe a potential vaccine candidate for the prevention of ASF and Pseudorabies.

## Background

African swine fever (ASF) is a highly contagious viral disease, a disease that must be reported to the World Organization for Animal Health (OIE) [1]. ASF was first reported in Kenya in 1921 [2], and then gradually spread to neighboring countries, in 2007–2017 to Georgia, Russia, Romania, and other European countries [3], and in 2018 to China, Mongolia, Vietnam, Cambodia, Laos, North Korea, and South Korea and other Asian countries [4, 5]. The disease is caused by the ASF virus (ASFV), a double-stranded DNA virus with a large genome (170–193 kb) [6]. ASFV has 151–167 open reading frames (ORFs) and encodes 54 structural proteins and more than 100 non-structural proteins [7–10]. ASFV is the only known DNA arbovirus, has 24 genotypes and 8 serotypes, and sole member of the *Asfarviridae* family [10, 11]. A species of soft tick (*Ornithodoros moubata*) is the natural host of ASFV. The virulent ASFV strains can cause acute hemorrhagic fever and lead to up to 100% mortality in domestic pigs and wild boar (*Sus scrofa*) [6, 12]. Currently, there is no commercial vaccine to prevent ASF, and the epidemic is still spreading.

At present, various types of vaccines are under development to prevent ASF, including inactivated vaccines, live attenuated vaccines, subunit vaccines, DNA vaccines, virus vector vaccines, etc. Studies have shown that either traditional inactivated vaccines alone or with new adjuvants can produce specific antibodies but has no protective effect [13, 14]. The single or combination of ASFV CD2v, P30, P54, and other subunit vaccines show some protective effects [15, 16]. The "cocktail" immunization by using adenovirus vector vaccines carrying EP402R Δ PRR, p54, p72, and so on, and the recombinant B646l (p72), EP153R and EP402R (CD2v) vaccinia virus vaccines can all induce strong humoral and cellular immunity [17, 18].

Cytotoxic T lymphocyte (CTL) immune response plays an important role in protecting ASFV [19]. Most DNA vaccines, such as a DNA vaccine expressing the ubiquitin (Ub)/CD2v/P54/P30 fusion protein, can effectively activate CTL immune response and provide partial protection after immunization in pigs [20, 21]. A vaccine that contains 80 ORFs of ASFV (Ba71V) fused with ubiquitin can provide 60% protection after immunization, and the specific CTL immune response can be induced after the virus challenge [22]. Natural attenuated strains or improved live viruses show good protective effects, but most live attenuated vaccines only had a protective effect on the homologous strains [23–26]. Besides, most live attenuated vaccines lack high-yield cell lines. ASFV mainly infects the host monocyte-macrophage system through megapinocytosis and cytophagocytosis mediated by grid protein, and the production is low in the primary monocytes/macrophages [27, 28]. Besides, attenuated ASFV can produce adverse side effects, such as lymphadenopathy, recurrent fever, chronic viremia, persistent chronic infections and the possibility of virulence recovery [23]. These adverse factors hamper their development as usable vaccines. In contrast, subunit vaccines, DNA vaccines, and viral vector-based vaccines show advantages in terms of safety and productivity and are likely better options to be developed for preventing ASF.

Pseudorabies virus (PRV), *Alphaherpesvirinae* subfamily of the *Herpesviridae*, is the pathogen of swine *Aujeszky's disease*, a double-stranded DNA virus with a genome size of 145 kb. Domestic pigs and wild boars are the natural hosts of PRV [29, 30]. PRV can infect pigs of all ages, but the symptoms appear different. Infection of PRV in adult pigs will lead to abortion and respiratory symptoms, while in piglets will lead to fatal encephalitis, which has caused huge economic losses in the global pig breeding industry [31]. Other mammals, such as rodents, can also be infected by PRV, and the host systemic inflammatory response, especially neuroinflammation, can cause severe itching and acute death in infected mice [32].

The PRV Bartha-K61 vaccine has been used to protect pigs from PRV infection. However, in recent years, a new PRV clade which belongs to the genotype II has been found in the pigs immunized with Bartha-K61, which belongs to genotype I. Thereby, the Bartha-K61 vaccine does not provide complete protection for the type II virus [30, 33] and new vaccines are needed to prevent the emergence of new epidemics. Several new vaccine strains, such as PRV TK^−^/gE^−^/gI^−^ (Fa) [34], JS-2012-ΔgE/gI [29], gE^−^/gI^−^/TK^−^ PRV (HeN1) [35], have all showed good protection capabilities, and PRV TK^−^/gE^−^/gI^−^ (Fa) has been commercially used for the prevention of pseudorabies. PRV has more than 70 ORFs that encode 70–100 proteins, but only about 50 proteins are contained in mature virus particles. Many genes are unnecessary for PRV replication, such as TK, gE, and gI, which can be replaced by foreign genes [36–38]. Therefore, PRV is often used as a viral vector to carry foreign genes to develop recombinant vaccines. Many PRV recombinant strains, such as JS-2012-ΔgE/gI-E2 (expressing classical swine fever virus E2 protein) [39], PRV SA215/VP2 (expressing parvovirus VP12 protein) [40], PRV-P12A3C (expressing foot-and-mouth disease virus P12A and 3C proteins) [41], show good immunogenicity and safety.

The outer envelope protein CD2v of ASFV, encoded by the EP402R gene, is highly similar to the host CD2 protein in structure and function and is the key protein that mediates the ASFV binding with red blood cells and causes their adsorption [42]. ASFV infection can inhibit the proliferation of peripheral blood lymphocytes in vitro, which can be rescued by the EP402R gene deletion [43]. Deletion of CD2v can strongly inhibit ASFV proliferation by 100–1000 times in lymphoid tissue and bone marrow, and ameliorate viremia [43]. Thus, CD2v is likely to be involved in the immune escape, tissue phagocytosis of ASFV, and immunosuppressive effects. Blocking CD2v may revoke the host immunosuppressive effect, thereby enhancing the host's antiviral ability. CD2v has been developed as subunit, DNA, and virus vector vaccines, to provides partial protection [15, 20, 44]. At present, there is no report on the recombinant multivalent vaccine that recombinantly expresses the ASFV gene using PRV as a vector. In this study, we constructed a recombinant pseudorabies virus, PRV-ΔgE/ΔgI/ΔTK-(CD2v), that expresses the ASFV CD2v protein by using the CRISPR/Cas9 technology, and its safety and ability to produce humoral and cellular immune responses were evaluated in mice to provide evidence for the future vaccine development to prevent both African swine fever and Pseudorabies.

## Materials and methods

### Cells, virus, and mouse

All cell culture reagents were purchased from Life Technologies (CA, USA) unless otherwise indicated. Human embryonic kidney (HEK 293 T) cells and Vero cells were cultured in Dulbecco's Modified Eagle Medium (DMEM) containing 10% FBS (GIBCO), 100 U/ml penicillin, 100 mg/ml streptomycin, and 5% CO_2_ at 37 °C. The peripheral blood lymphocytes of mice were cultured in RPMI 1640 medium. EXPi293 cells were purchased from Thermofisher (CA, USA) and cultured in a constant temperature shaker with OPM-293 CD03 medium under 8% CO_2_, 37 °C, 220 rpm/min. PRV was obtained from Fujian Academy of Agricultural Sciences (Fujian, China) and expanded in Vero cells. The viral titer was determined by the Karber method as follows: 2 × 10^4^ Vero cells in 90 μl DMEM were mixed with 10 μl virus solution and plated onto 96-well plates for tenfold gradient dilution, and 8 multiple wells were set for each dilution gradient. The cytopathic effect was observed every 24 h until no change appeared in any well. The titer's calculation formula is lgTCID_50_ = L-D (S-0.5), where L is the logarithm of the highest dilution, D is the difference between the logarithm of dilution, and S is the sum of the proportion of positive wells.

Four-week-old SPF-ICR mice were purchased from Wushi Experimental Animal Trade Co., Ltd (Jiangsu, China). The animal experiments were performed under the Guide for the Care and Use of Laboratory Animals approved by Fujian Provincial Office for Managing Laboratory Animals and were overseen by the Fujian Normal University Animal Care and Use Committee (SYXK: 2015-0004).

### Plasmids and viruses construction

Construction of the knockout (KO) plasmids used the pX459 plasmid vector (purchased from Addgene, MA, USA, digest with BpiI (37 °C, 15 min) (purchased from Thermo Fisher Scientific, MA, USA)) and sgRNA sequences (Table 1) targeting the PRV gE, gI and TK genes according to the website (https://www.e-crisp.org/e-crisp/designcrispr.html). After transformation into DH5α *E. coli*, the recombinant plasmids were isolated and verified by sequencing.

**Table 1:** The sgRNA sequences target PRV-Fa gE, gI, TK genes

A. Primers name	B. sgRNA sequence 5′–3′
PRV-TK-sgRNA-F	5′-caccgTGCCCGAGCCGATGGCGTGC-3′
PRV-TK-sgRNA-R	5′-aaacGCACGCCATCGGCTCGGGCAc-3′
PRV-gE-sgRNA-F	5′-caccgGCCGGCGACGATGACCTCGA-3′
PRV-gE-sgRNA-R	5′-aaacTCGAGGTCATCGTCGCCGGCc-3′
PRV-gI-sgRNA-F	5′-caccgCGCGGGGTCGTACGTGCTGC-3′
PRV-gI-sgRNA-R	5′-aaacGCAGCACGTACGACCCCGCGc-3′

The eukaryotic expression vector pcDNA3.4-(N-CD2v)-His was constructed using pcDNA3.4 (purchased from Addgene, MA, USA). The extracellular region of CD2v (46–615 bp, encoding 16-205aa) synthesized by the Wuhan GeneCreate Biological Engineering (Hubei, China) was inserted into pcDNA3.4 and fused with the His-tag at the C-terminus. The full-length CD2v gene (based on pig/HLJ/2018 [1] strain) was synthesized by Wuhan GeneCreate Biological Engineering (Hubei, China) and used to make pcDNA3.1-EGFP-Flag-CD2v-Flag. The recombinant vectors were transformed into *E. coli DH5α* (DE3). All plasmids were extracted using a kit (DP118-02) purchased from TIANGEN BIOTECH (Beijing, China).

To generate gene-deleted PRVs, the KO plasmids targeting PRV gE, gI, TK were transfected into 5 × 10^5^ HEK 293 T cells. Six h after transfection, 1 × 10^5^ TCID_50_ PRV-Fa was added. The virus culture was harvested when the cytopathic effect reached 90% or more. The sgRNA-induced mutation was determined by PCR and sequencing by the primers as shown in Table 2. Four rounds of plaque purification were performed in Vero cells to obtain pseudorabies gE, gI double gene deletion strain PRV-ΔgE/ΔgI and gE, gI, TK triple gene deletion strain PRV-ΔgE/ΔgI/ΔTK.

**Table 2:** Primers for verifying viral genes mutation

A. Primers name	B. Primers sequence 5′–3′
PRV-TK-KO-F	5′-TCGTAGAAGCGGTTGTGG-3′
PRV-TK-KO-R	5′-CGACCAGGACGAACAGG-3′
PRV-gE-KO-F	5′-AAAAGGTGGTGTTTGCATAATT-3′
PRV-gE-KO-R	5′-TCGGTGGTGATGTAGAACG-3′
PRV-gI-KO-F	5′-GTGGGCGTGTGCGTCTA-3′
PRV-gI-KO-R	5′-CGGACGGAGATAAAACGC-3′

To generate recombinant PRV (PRV-ΔgE/ΔgI/ΔTK-(CD2v)), 5 μg of plasmid and 1 μg of the homologous recombinant fragment were co-transfected into HEK 293 T cells. Six h after transfection, 5 × 10^5^ TCID_50_ of PRV-ΔgE/ΔgI was added. The virus culture was harvested when 90% of the cells were assured of having cytopathic effects. Finally, the recombinant PRV-ΔgE/ΔgI/ΔTK-(CD2v) strain expressing ASFV CD2v was amplified and purified in Vero cells by four rounds of phagocytosis.

### Immunofluorescence

HEK 293 T cells (2 × 10^5^) were cultured for 24 h, and transfected with pcDNA3.1-EGFP-Flag-CD2v-Flag or infected with 5 × 10^4^ TCID_50_ viruses for 36 h. The cells were fixed with 1 ml 4% paraformaldehyde, penetrated with 1 ml 0.1% Triton X-100 (Sangon Biotech, Shanghai, China), incubated by mouse anti-Flag monoclonal antibody (TransGen Biotech, China) followed by Goat anti-mouse IgG H & L (Alexa fluor ® 488) preadsorbed (ab150117), and the images were obtained by scanning with ZEISS LSM700 microscope.

### Western blot analysis

HEK 293 T cells were cultured for 24 h, and transfected with the plasmid pcDNA3.1-EGFP-Flag-CD2v-Flag or infected with 1 × 10^6^ TCID_50_ of PRV-ΔgE/ΔgI/ΔTK-(CD2v). Samples were collected 48 h after transfection or viral infection. The protein concentration was determined by a BCA protein concentration test kit (from Beyotime Biotechnology, Shanghai, China)). Sixty μg of proteins were loaded for SDS–polyacrylamide gel electrophoresis and transferred to the PVDF membrane. The membrane was sealed with 5% skim milk at room temperature for 2 h, then incubated with the mouse anti-Flag monoclonal antibody (1:1000 dilution, ProteinFind® Anti-DYKDDDDK Mouse Monoclonal Antibody HT201-01 (TransGen Biotech, (Beijing, China)) or GAPDH (14C10) Rabbit mAb (Cell Signaling Technology, USA) in 5% skimmed milk plus 100 mg/L NaN_3_ at 4 °C for 8 h, washed with TBST buffer for 3 times, followed by incubation with secondary antibody IRDye 800cw donkey anti-mouse IgG (LI-COR) (1:10,000) at room temperature for 2 h. The membrane was washed with TBST and visualized by using LI-COR Odyssey infrared fluorescence scanning imager.

### Virus growth and pathogenicity analyses

To determine the growth kinetics of the virus, 5 × 10^5^ Vero cells in 2 ml DMEM were seeded onto each well of 6-well plates, 1 × 10^3^ TCID_50_ viruses was added into each well, and the sample was collected at 12, 24, 36, 48 h post-infection, and virus titer was calculated by the Karber method.

The growth of plaques in Vero cells was observed by doubled-blinded crystal violet staining. Vero cells (5 × 10^5^/each well) in the 6-well plates were infected with PRV-Fa, PRV-ΔgE/ΔgI/ΔTK, PRV-ΔgE/ΔgI/ΔTK-(CD2v) of 50 TCID_50_ for 48 h, and fixed with 4% paraformaldehyde for 30 min, stained with 2.5% crystal violet. Ten plaques were randomly selected to measure their areas under the microscope.

For the pathogenicity test, 4-week-old SPF grade ICR mice were fed adaptively for 1 week. The survival of mice was observed by injecting 5 × 10^5^ TCID_50_ into the right hind leg muscle, and the survival curve was drawn accordingly.

### (N-CD2v)-His purification

The EXPi 293 cells were transfected with pcDNA3.4-(N-CD2v)-His plasmid, collected after 7 days of culture, and lysed with RIPA (about 500 μl of RIPA per 1 × 10^7^ cells). The (N-CD2v)-His protein was enriched with a nickel column and eluted with Elution Buffer (300 mM imidazole, 1 mM PMSF, 25 μl β-mercaptoethanol). The eluate was vacuum dried, dissolved in pH7.4 PBS, and used for SDS polyacrylamide gel electrophoresis. The purity of the recombinant protein was determined by Coomassie staining.

### Virus copy analyses in mice

When the control PRV-Fa-infected mice group began to scratch (about 72 h postinoculation), the mice in all groups were euthanized by CO_2_ inhalation. The heart, brain, lung, liver, spleen and kidney tissues were collected, and the corresponding DNA was extracted. Real-time quantitative polymerase chain reaction (qPCR) was used to determine viral genome copies in infected cells and tissues. The PRV UL42 gene was used for standard control and was inserted into the pCDH plasmid to make pCDH-UL42. The UL42 gene was also amplified from the genomic DNA of the PRV-Fa strain using the forward primer 5′-ATGTCGCTGTTCGACGAC-3′ and the reverse primer 5′-TTAGAATAAATCTCCGTAGGCG-3′. Viral and tissue genomic DNA was extracted and purified by using a DNA Extraction Kit DP304 (TIANGEN BIOTECH (BEIJING)), and the PCR products were purified using FastPure Gel DNA Extraction Mini Kit DC301 (Vazyme Biotech, Jiangsu, China).

The virus copy number was also examined in feces. The feces of mice were picked up every 24 h after the virus challenge. One gram of feces was mixed with 5 ml of phosphate buffer saline (PBS) and soaked for 2 h, and the supernatant was collected by centrifugation at 3000 g for 10 min and used for extracting the genome DNA for qPCR determination of virus nucleic acid copies. The primers were: forward (5′-AACGTCACCTTCGAGGTGTA-3′) and reverse (5′-AGTCTGAACTCGTGCTTG-3′).

The virus copy number was calculated as follows: the average molecular weight (Dalton) of pCDH-UL42 = base number × 600 (Base pair average molecular weight), the length of pCDH-UL42 = 8542 bp (pCDH) + 1158 bp (PRV-UL42) = 9700 bp, and the copy number of 1 ng pCDH-UL42 = 6.02 × 10^23^ × (1 × 10^–9^/(9700 × 600)) ≈1.0 × 10^8^. The standard curve was generated using the copy number of pCDH-UL42 as the ordinate, and the CT values determine the corresponding CT value as the abscissa and the copy number of the PRV genome.

### Cytokine expression analyses in mice

The heart, brain, lung, liver, spleen and kidney tissues from the virus-infected mice were collected, and the total RNA was extracted. The RNA was extracted using Trizol Gamma Reagent (Thermo Fisher Scientific, CA, USA). cDNA was generated by reverse transcription using MonScript™ RTII All-in-One Mix with dsDNase Kit (Mona Biotech, Jiangsu, China) according to the manufacturer's instruction. The RNA expression of cytokines (see Table 3 for cytokine primers) was analyzed by reverse transcription qPCR.

**Table 3:** Primers for RT-qPCR

A. Primers name	B. Primers sequence 5′–3′
MUS-IL6-q-PCR-F	5′-CTGCAAGAGACTTCCATCCAG-3′
MUS-IL6-q-PCR-R	5′-AGTGGTATAGACAGGTCTGTTGG-3′
MUS-IL1β-q-PCR-F	5′-GAAATGCCACCTTTTGACAGTG-3′
MUS-IL1β-q-PCR-R	5′-TGGATGCTCTCATCAGGACAG-3′
MUS-TNFα-q-PCR-F	5′-CAGGCGGTGCCTATGTCTC-3′
MUS-TNFα-q-PCR-R	5′-CGATCACCCCGAAGTTCAGTAG-3′
MUS-IFNβ-q-PCR-F	5′-AGCTCCAAGAAAGGACGAACA-3′
MUS-IFNβ-q-PCR-R	5′-GCCCTGTAGGTGAGGTTGAT-3′

For testing interferon-γ (IFNγ), on day 7 after mice second immunization, peripheral blood lymphocytes (PBMCs) were obtained by collecting 1 ml of cardiac blood following the treatment with red cell lysate (Beyotime Biotechnology, China). PBMCs (1 × 10^6^) were cultured for 12 h and treated with 10 μg recombinant protein (N-CD2v)-His for 72 h. The cells were then harvested, and the IFNγ transcription level was measured by qRT-PCR. The primers used for analyses were: IFNγ-Forward (5′-GCCACGCACAGTGATTGA-3′); IFNγ-Reverse (5′-TGCTGATGCCTGATTTGTCTT-3′).

### Flow cytometry analysis of CD3/CD4/CD8/CD69 peripheral blood lymphocytes

When the control PRV-Fa group began to scratch (about 72 hpi), about 300 μl of blood was collected from the mouse orbit for analyses of CD3^+^, CD3^+^CD4^+^, CD3^+^CD8^+^, CD3^+^CD69^+^, CD4^+^CD69^+^, CD8^+^CD69^+^ peripheral blood lymphocytes by flow cytometry. Two hundred μl of blood was mixed with 1 μl of antibody (BV421 Hamster Anti-Mouse CD3ε, FITC Rat Anti-Mouse CD4, PerCP-CY 5.5 Rat Anti-Mouse CD8α, PE Hamster Anti-Mouse CD69 (BD pharmaceuticals, CA, USA) for 30 min in the dark at 25 °C, incubated with 600 μl of RBC lysate (Shanghai Biyuntian Biotechnology, China) for 5 min. The cells were collected by centrifugation and suspended in PBS containing 2% FBS followed by flow cytometry analyses (BD FACSymphony™ A5, BD Bioscience, CA, USA).

#### Immunization and challenge

Five-week-old SPF mice (ICR) were injected with either PRV-ΔgE/ΔgI/ΔTK or PRV-ΔgE/ΔgI/ΔTK-(CD2v) (100 μl each, 1 × 10^5^ TCID_50_) via the *i.m.* route, and strengthened by the second immunization one week later. Seven days later, the mice were challenged by PRV-Fa with 5 × 10^5^ TCID_50_. The control group was injected with 100 μL DMEM.

#### Enzyme-linked immunosorbent assay (ELISA) for Flag IgG

About 300 μl of blood was collected from the mouse orbit 0, 7 and 14 days after the first immunization. The supernatant was collected by centrifugation at 3000 rpm/min for 5 min after the blood was placed at room temperature for 10 min, and used for detecting the Flag IgG by ELISA using SBJ-M0915-96 T Flag-tag AB ELISA Kit (SenBeiJia Biological Technology, Jiangsu, China).

#### Hematoxylin–Eosin staining

Mouse tissues were fixed with 4% paraformaldehyde for 12 h. The samples were treated with 30% ethanol, 50% ethanol, 70% ethanol, 90% ethanol, 95% ethanol and anhydrous ethanol for 30 min each, followed by transparent treatment with the mixture of ethanol and xylene (1:1), and xylene to replace the ethanol in the tissues. The tissues were treated with a mixture of paraffin and xylene (1:1) overnight, then embedded into the paraffin. The tissues were cut to the 7 μm slices followed by staining with hematoxylin and eosin, and the morphological changes were checked by Zeiss Axio Imager A2/D2/m2/Z2.

#### Data statistical analysis and image processing

Images for Western blotting, H&E staining, crystal violet staining and immunofluorescence were processed by Adobe Illustrator CS6, and the sequence data were analyzed by BioEdit. All experiments were repeated at least 3 times independently. Unpaired *t*-test or two-way ANOVA (Graphpad Prism 5.0, Graphpad Software, San Diego, CA, USA) was used to analyze data differences between groups. Data are presented as the mean ± SEM in the same treatment.

## Results

### Generation of the recombinant pseudorabies virus PRV-ΔgE/ΔgI/ΔTK-(CD2v)

**Fig. 1:**
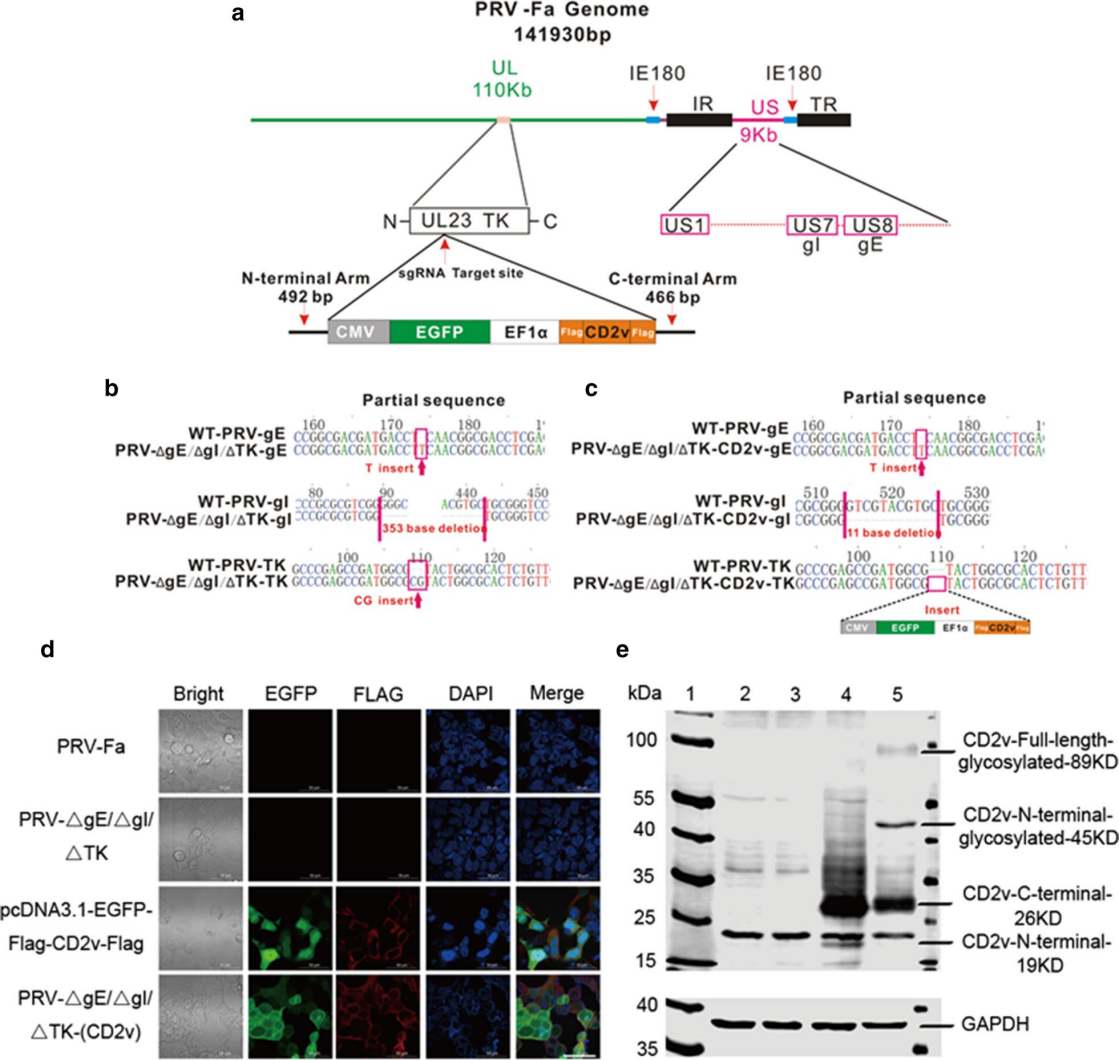
Construction of recombinant and attenuated strains. **a** The recombinant fragment was inserted between the 108th and 109th bases at the TK gene's N-terminus. The N-terminal homologous arm was 492 bp, and the C-terminal homologous arm was 466 bp. EGFP was driven by the CMV promoter and Flag-CD2v-Flag by the EF1α promoter. **b** The attenuated strain of PRV-ΔgE/ΔgI/ΔTK was made by insertion of a single base (T) in the US8 (gE) gene, deletion of base (353 bp) in the US7 (gI) gene, and insertion of two bases (GC) in the UL23 (TK) gene. **c** The attenuated strain of PRV-ΔgE/ΔgI/ΔTK-(CD2v) was made by insertion of single base (T) in the US8 (gE) gene, deletion of 11 bases (11 bp) in the US7 (gI) gene, insertion of CMV-EGFP-EF1-Flag-CD2v-Flag in the UL23 (TK) gene (which disrupted the TK gene). **d** Immunofluorescence showed that CD2v was expressed in HEK 293 T cells transfected with pcDNA3.1-EGFP-Flag-CD2v-Flag plasmid or infected with PRV-ΔgE/ΔgI/ΔTK-(CD2v) (red is CD2v labeled with Flag) and white scale represents 50 μm. **e** Western blotting showed that CD2v bands (89 KD full-length glycosylation band, 45 KD N-terminal glycosylation band, 26 KD C-terminal band, 19 KD N-terminal band) were detected in HEK 293 T cell lysates transfected with pcDNA3.1-EGFP-Flag-CD2v-Flag plasmid or infected with PRV-ΔgE/ΔgI/ΔTK-(CD2v). **e** Lane 1: protein marker, Lane 2: control, Lane 3: pcDNA3.1 transfected group, Lane 4: pcDNA3.1-EGFP-Flag-CD2v-Flag transfected group, Lane 5: PRV-ΔgE/ΔgI/ΔTK-(CD2v)-infected group

The recombinant pseudorabies virus PRV-ΔgE/ΔgI/ΔTK-(CD2v) expressing ASFV CD2v protein was constructed by homologous recombination using the CRISPR/Cas9 technology. As shown in Fig. 1a, the EGFP was driven by the CMV promoter, and the CD2v was driven by the EF1α promoter. The recombinant fragment was inserted into the N-terminus of the PRV-ΔgE/ΔgI UL23 (TK) gene to construct PRV-ΔgE/ΔgI/ΔTK-(CD2v), in which the TK gene was disabled. The PRV-ΔgE/ΔgI/ΔTK and PRV-ΔgE/ΔgI/ΔTK-(CD2v) mutations were sequenced as shown in Fig. 1b, c. CD2v was successfully expressed in HEK 293 T cells, as shown by immunostaining (Fig. 1d) and Western blotting (Fig. 1e). CD2v can be expressed in HEK 293 T cells either by pcDNA3.1-EGFP-Flag-CD2v-Flag transfection or PRV-ΔgE/ΔgI/ΔTK-(CD2v) infection. Transfection with pcDNA3.1-EGFP-Flag-CD2v-Flag CD2v mainly shows the expression of the 26 KD C-terminus and 19 KD N-terminus cleaved proteins, while infection with PRV-ΔgE/ΔgI/ΔTK-(CD2v) resulted in the expression of the glycosylated 89 KD full length and glycosylated 45 KD N-terminus fragment, and the 26 KD C-terminus fragment (Fig. 1e). As expected, CD2v was not expressed in HEK 293 T cells transfected with pcDNA3.1 or infected with PRV-ΔgE/ΔgI/ΔTK. These results indicate that the recombinant pseudorabies strain PRV-ΔgE/ΔgI/ΔTK-(CD2v) was successfully constructed and CD2v processing is independent of ASFV infection.

### The recombinant strain can proliferate well in vitro and is safe for mice

**Fig. 2:**
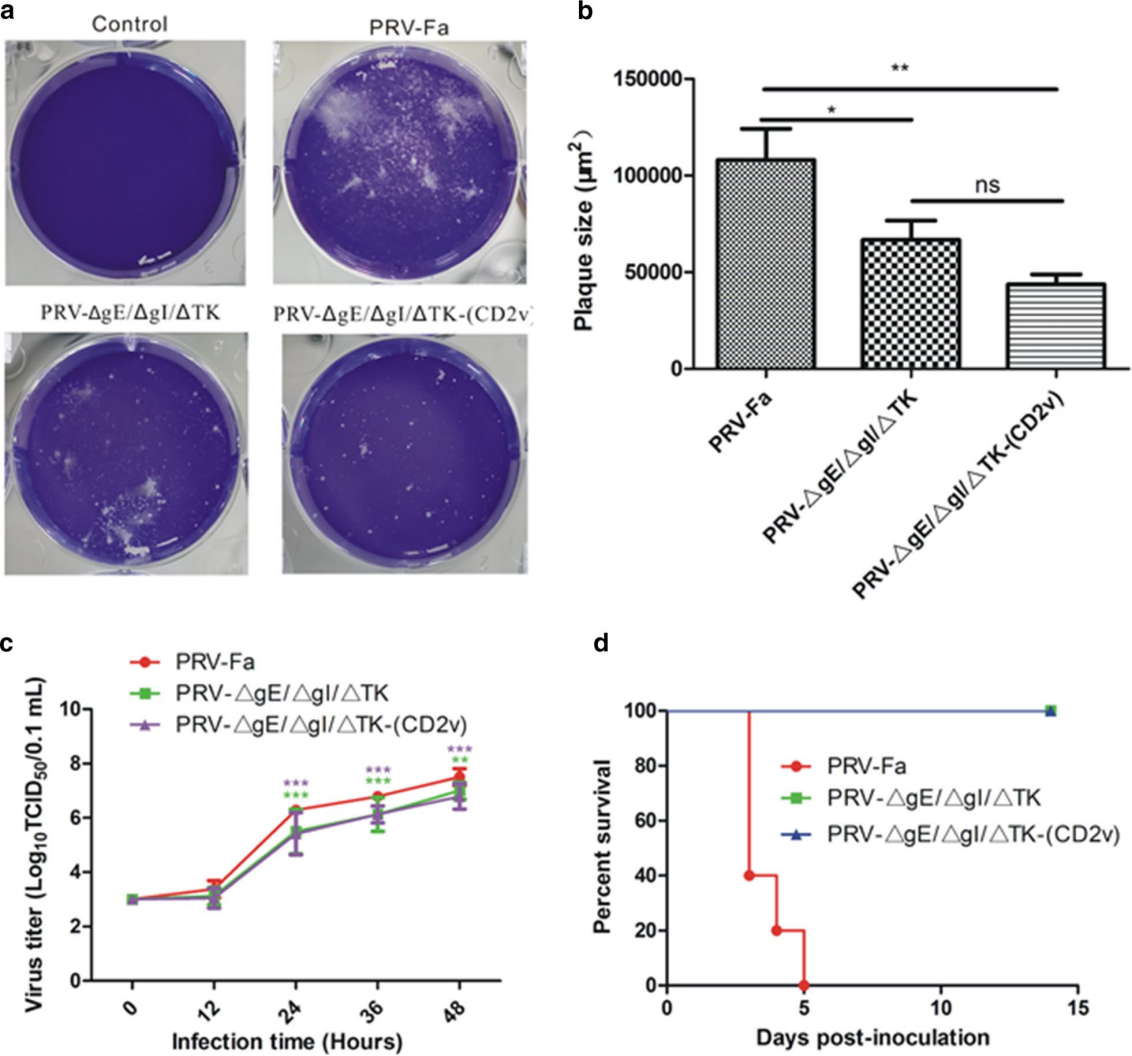
The insertion of CD2v did not change the proliferation and virulence of PRV-ΔgE/ΔgI/ΔTK. **a** The 50 TCID50 virus was inoculated into 5 × 10^5^ Vero cells and cultured for 48 h. The growth of plaques in Vero cells was observed by crystal violet staining. **b** Statistical results of the plaque area. **c** 1 × 10^3^ TCID50 viruses were inoculated into 5 × 10^5^ Vero cells to allow virus proliferation, and the virus sample was collected at 12 h, 24 h, 36 h, 48 h. The virus titer was calculated by the Karber method to draw a one-step growth curve. **d** Five-week-old SPF ICR mice were injected (i.m) with 5 × 10^5^ TCID50 viruses into the right hind leg to observe mice's survival daily, and the survival curve was drawn (n = 15/each group). Unpaired t-test or two-way ANOVA was performed by GraphPad Prism 5.0, GraphPad Software (San Diego, CA, USA), *p < 0.05, **p < 0.01, ***p < 0.001, ns (not significant)

The proliferation of PRV-ΔgE/ΔgI/ΔTK-(CD2v) was tested in the infected Vero cells. PRV-ΔgE/ΔgI/ΔTK-(CD2v) and PRV-ΔgE/ΔgI/ΔTK were far less capable of infecting the Vero cells compared with PRV-Fa at the designated concentrations (Fig. 2a–c). The proliferative ability between PRV-ΔgE/ΔgI/ΔTK-(CD2v) and PRV-ΔgE/ΔgI/ΔTK was not significantly different in Vero cells (Fig. 2c), suggesting that CD2v does not change the virus growth dynamics in vitro. Then, we used mice to evaluate the safety of the recombinant strains. As shown in Fig. 2d, all mice in the PRV-Fa-infected group died on the 5th day of inoculation, while the mice infected with either PRV-ΔgE/ΔgI/ΔTK, or PRV-ΔgE/ΔgI/ΔTK-(CD2v) did not die until the 14th day. We observed severe pruritus in the PRV-Fa group on the 3rd day following inoculation, but not in PRV-ΔgE/ΔgI/ΔTK or PRV-ΔgE/ΔgI/ΔTK-(CD2v) infected groups (Additional file 1: Figure S1), suggesting that the insertion of CD2v does not change the pathogenicity of PRV-ΔgE/ΔgI/ΔTK.

**Fig. 3:**
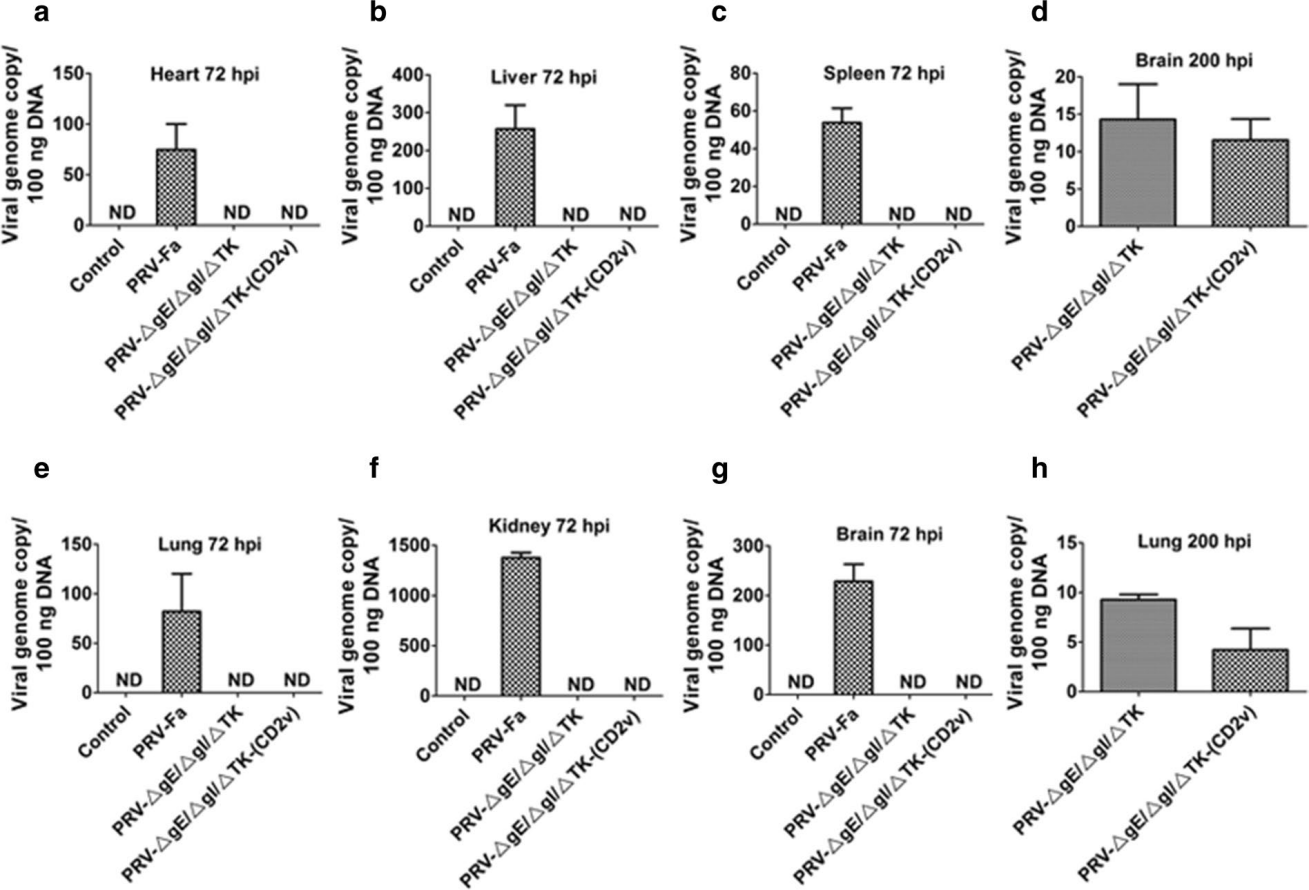
The virus DNA copies present in the various organs of mice inoculation by viruses, including heart (**a**), liver (**b**), spleen (**c**), lung (**e**), kidney (**f**), and brain (**g**) at 72 hpi. The virus DNA copies present in the various organs of mice inoculation by viruses, including the brain (**d**) and lung (**h**) at 200 hpi. Five-week-old SPF-ICR mice were inoculated with 1 × 10^5^ TCID50 viruses by intramuscular injection in the right hind leg. The control group was injected with 100 μL DMEM. When the PRV-Fa infected group started to scratch (about 72 hpi) or extended the infection time to 200 h, the mice of different viral treated and untreated groups were sacrificed, and the organs were collected for genomic DNA extraction, and 100 ng genome was used to perform qPCR analyses to determine viral nucleic acid copy number (n = 5/each group), ND (not detect)

The virulent effect of CD2v was also tested by lethality, tissue pathology, expression of inflammatory factors and tissue inflammation in the mice infected with PRV-Fa, PRV-ΔgE/ΔgI/ΔTK, PRV-ΔgE/ΔgI/ΔTK-(CD2v) at the designated concentrations. By qPCR and qRT-PCR, we examined the viral copy number and the IL6, Tumor necrosis factor-α (TNFα), Interleukin1-β (IL1β); Interferon-β (IFNβ) transcription levels in the brain, heart, lung, liver, kidney, and spleen tissues of infected mice. The viral genome DNA was detected in all tissues in PRV-Fa-infected mice, but not in PRV-ΔgE/ΔgI/ΔTK or PRV-ΔgE/ΔgI/ΔTK-(CD2v) infected group at 72 hpi (Fig. 3a–f). When the infection time was extended to 200 h, viral nucleic acid was detected in the brain and lungs of mice infected with PRV-ΔgE/ΔgI/ΔTK or PRV-ΔgE/ΔgI/ΔTK-(CD2v) (Fig. 3g–h). However, the copy number of the virus is very low compared with the PRV-Fa infection at 72 hpi. These results indicate that the insertion of CD2v does not change the histopathology of PRV-ΔgE/ΔgI/ΔTK. The sensitivity of qPCR for detecting virus copies is shown in Additional file 2: Figure S2, S2A is the pCDH-UL42 plasmid map, Additional file 2: Figure S2B is the amplification curve, and Additional file 2: Figure S2C is the calibration equation as Y = −0.2425X + 9.2678 (R^2^ = 0.994), Additional file 2: Figure S2D is a gel electrophoresis image of the qPCR product. According to Additional file 2: Figure S2B and S2D, the limit for virus copy detection by qPCR is between 10 to 100.

**Fig. 4:**
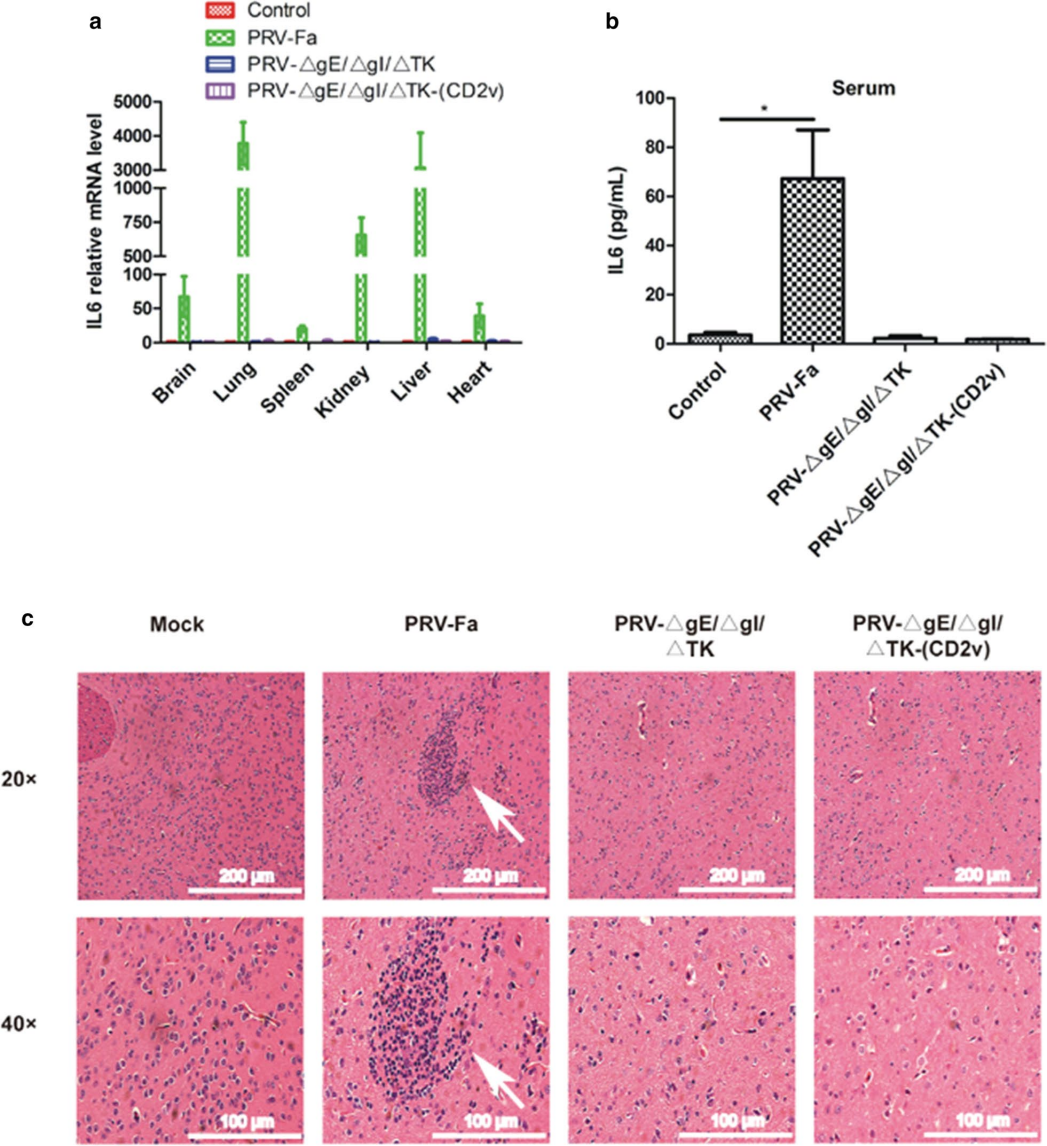
The recombinant and attenuated strains did not cause systemic inflammation in mice. **a** Five-week-old SPF-ICR mice were inoculated with 1 × 10^5^ TCID50 viruses by intramuscular injection in the right hind leg. The control group (Mock) was injected with 100 μL DMEM. When the PRV-Fa-infected mouse group was found to start scratching (about 72 hpi), the surviving mice's organs were collected, 10 mg tissues were used to extract RNA, 100 ng cDNA generated from reverse transcription were used for qPCR to examine IL6 transcription. **b** The serum IL-6 was detected by ELISA after about 200 μl blood was collected from the orbit of control or virus-infected mice. **c** H & E staining showed that the infection of PRV-Fa could lead to the infiltration of immune cells in the brain of mice. The black arrow in the figure indicated the immune cell infiltration. Unpaired *t*-test was performed by GraphPad Prism 5.0, GraphPad Software (San Diego, CA, USA), *p < 0.05 (n = 5/each group)

PRV-Fa infection can lead to a large increase in the IL6 mRNA expression, whereas inoculation of PRV-ΔgE/ΔgI/ΔTK or PRV-ΔgE/ΔgI/ΔTK-(CD2v) did not cause up-regulation of IL6 expression (Fig. 4a). But, inoculation of each of these 3 virus strains did not cause changes in TNFα, IL1β, IFNβ transcription levels (data not shown). Consistently, the ELISA data showed a large increase in the IL6 protein in the serum of PRV-Fa infected mice, but not in the PRV-ΔgE/ΔgI/ΔTK or PRV-ΔgE/ΔgI/ΔTK-(CD2v) group as compared to the control uninfected group (Fig. 4b). H & E staining showed that PRV-Fa induced inflammatory cells' infiltration in the brain, but not in the other two infected groups (Fig. 4c). All the above data indicate that CD2v expression does not change the virulence of PRV-ΔgE/ΔgI/ΔTK, and PRV-ΔgE/ΔgI/ΔTK-(CD2v) is as safe as PRV-ΔgE/ΔgI/ΔTK.

### Recombinant strains can induce specific humoral and T cell immune responses in a mouse model

**Fig. 5:**
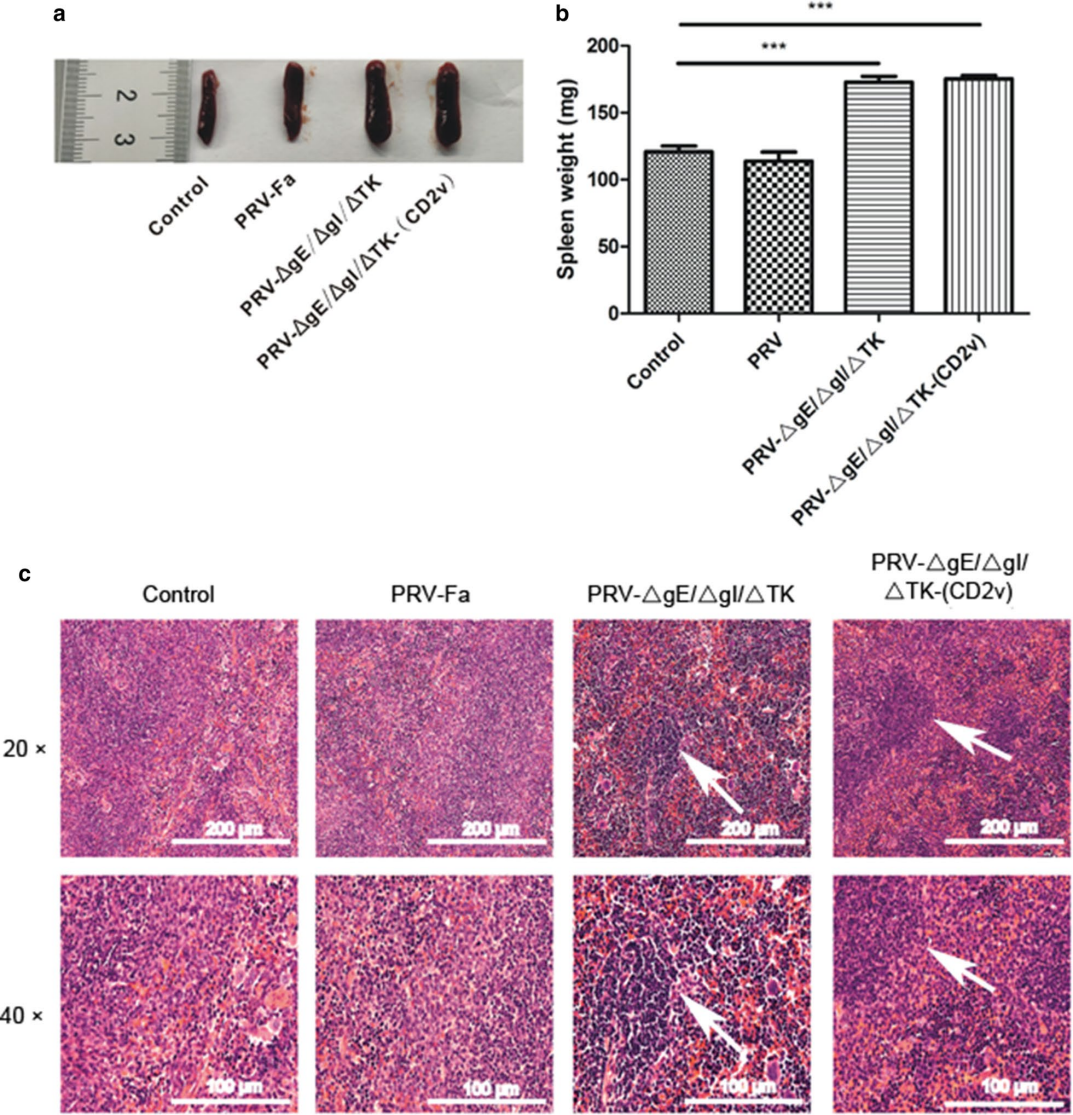
Both PRV-ΔgE/ΔgI/ΔTK and PRV-ΔgE/ΔgI/ΔTK-(CD2v) can activate the immune responses and cause the proliferation of immune cells leading to splenomegaly. **a** The spleen of 72 hpi mice inoculated with PRV-ΔgE/ΔgI/ΔTK and PRV-ΔgE/ΔgI/ΔTK-(CD2v) was swollen obviously. **b** Spleen weight statistics. **c** H & E staining showed that the spleen of PRV-ΔgE/ΔgI/ΔTK and PRV-ΔgE/ΔgI/ΔTK-(CD2v) infected groups had a large number of immune cell accumulation, as shown by the black arrow. Unpaired *t*-test was performed by GraphPad Prism 5.0, GraphPad Software (San Diego, CA, USA), ***p < 0.001 (n = 5/each group)

**Fig. 6:**
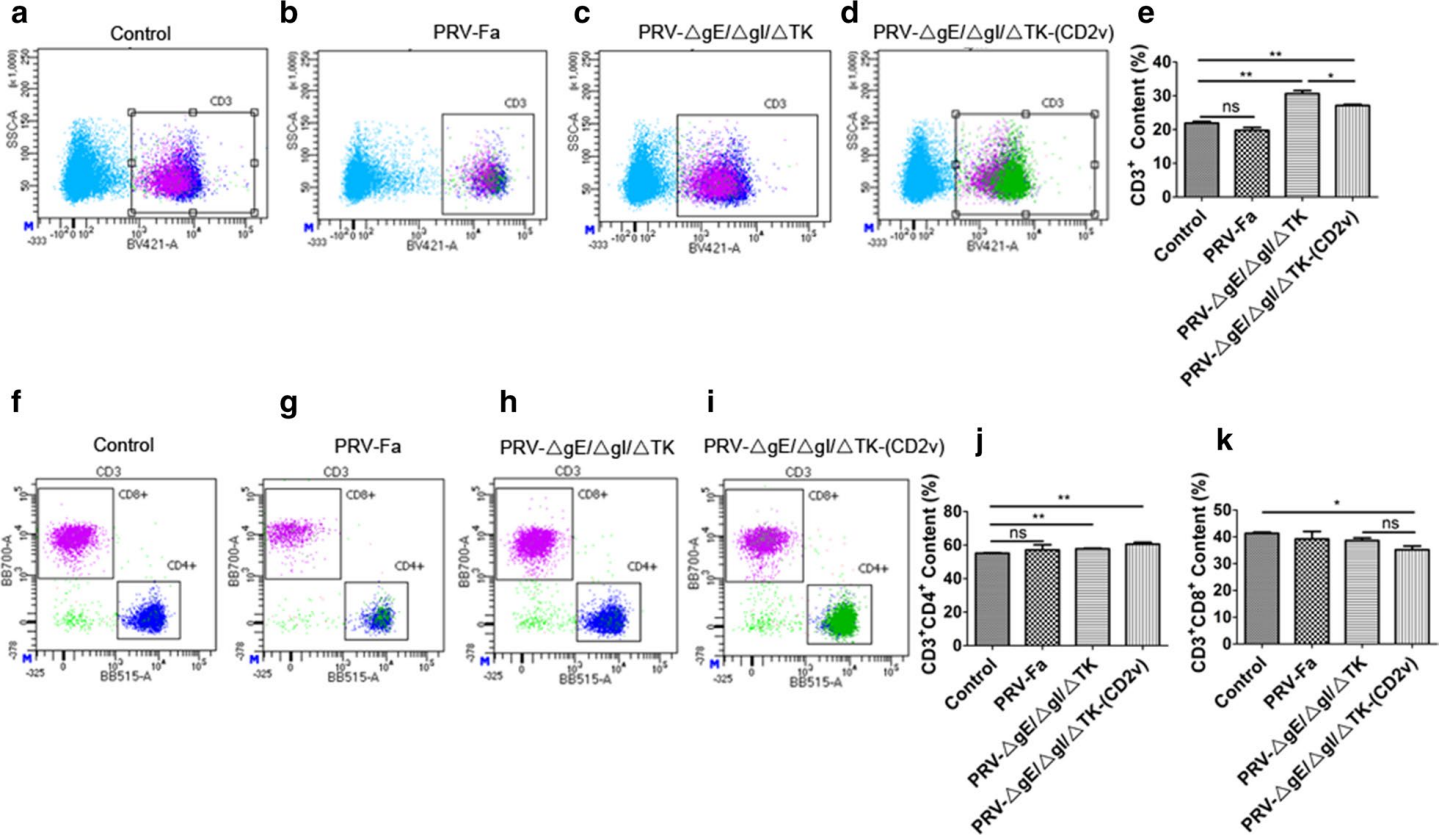
PRV-Fa, PRV-ΔgE/ΔgI/ΔTK and PRV-ΔgE/ΔgI/ΔTK-(CD2v) inoculation led to T cell proliferation. **a**–**d** The result of CD3^+^ T cell flow analysis. **f**–**i**: The result of CD3^+^CD4^+^, CD3^+^CD8^+^ T cell flow analysis. The data showed the changes of the percentage of CD3^+^ (**e**), CD3^+^CD4^+^ (**j**), CD3^+^CD8^+^ (**k**) T cells in total PBMCs. Five-week-old SPF-ICR mice were inoculated with 1 × 10^5^ TCID50 viruses by intramuscular injection in the right hind leg. The control group was injected with 100 μL DMEM. When the PRV-Fa-infected group started to scratch (about 72 hpi), the target cells were collected and analyzed by flow cytometry. Unpaired *t*-test was performed by GraphPad Prism 5.0, GraphPad Software (San Diego, CA, USA), *p < 0.05, **p < 0.01 (n = 5/each group)

We evaluate the possible immune effects of the recombinant virus strains. The spleen was significantly heavier in the mice infected by PRV-ΔgE/ΔgI/ΔTK or PRV-ΔgE/ΔgI/ΔTK-(CD2v) than that of the control untreated group (Fig. 5a,b). There was no difference in spleen weight between the immunized and control groups at 200 hpi (Additional file 3: Figure S3). H & E staining showed a significant infiltration of lymphocytes in spleen tissues of PRV-ΔgE/ΔgI/ΔTK or PRV-ΔgE/ΔgI/ΔTK-(CD2v) infected mice, but no difference was seen between the PRV-Fa group and control group (Fig. 5c). The percentage of CD3^+^, CD3^+^CD4^+^, CD3^+^CD8^+^ cells in PBMCs of mice is shown in Fig. 6. The percentage of CD3^+^ T cells in mouse PBMCs increased significantly by the inoculation with PRV-ΔgE/ΔgI/ΔTK or PRV-ΔgE/ΔgI/ΔTK-(CD2v) (*P* < *0.01*, *P* < *0.01*) (Fig. 6e). However, compared with PRV-ΔgE/ΔgI/ΔTK, PRV-ΔgE/ΔgI/ΔTK-(CD2v) has a weaker ability to induce T cell proliferation in the early stage of infection (72 hpi) (Fig. 6e). Further analysis of CD3^+^CD4^+^ and CD3^+^CD8^+^ T cell subtypes found that recombinant strain could inhibit CD3^+^CD8^+^ T cells' proliferation (Fig. 6k, Additional file 4: Figure S4I), suggesting that CD2v can interfere with the proliferation of T cells in response to mitogens. However, the recombinant strain does not affect CD3^+^ T cells' proliferation in the late stage of infection (200 hpi) (Additional file 5: Figure S5D).

**Fig. 7:**
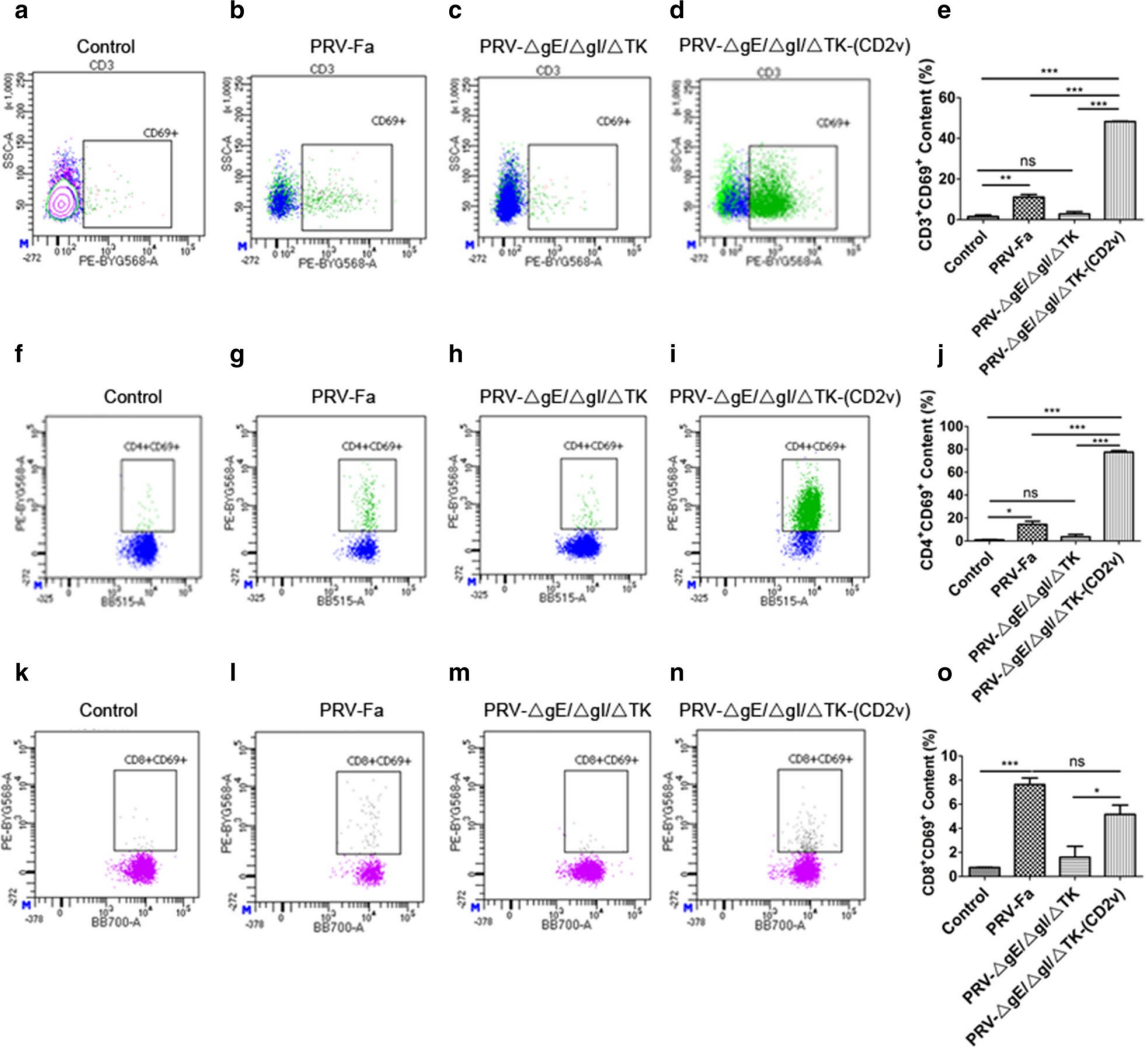
PRV-Fa, PRV-ΔgE/ΔgI/ΔTK and PRV-ΔgE/ΔgI/ΔTK-(CD2v) inoculation led to T cells' activation. **a**–**d** The result of CD3^+^CD69^+^ T cell flow analysis. **f**–**i**: The result of CD4^+^CD69^+^ T cell flow analysis. **k**–**n**: The result of CD8^+^CD69^+^ T cell flow analysis. The data showed the changes of the percentage of CD3^+^CD69^+^ (**e**), CD4^+^CD69^+^ (**j**), CD8^+^CD69^+^ (**o**) T cells in total PBMCs. Five-week-old SPF-ICR mice were inoculated with 1 × 10^5^ TCID50 viruses by intramuscular injection in the right hind leg. The control group was injected with 100 μL DMEM. When the PRV-Fa-infected group started to scratch (about 72 hpi), the target cells were collected and analyzed by flow cytometry. Unpaired t-test was performed by GraphPad Prism 5.0, GraphPad Software (San Diego, CA, USA), *p < 0.05, **p < 0.01, ***p < 0.001, (n = 5/each group)

We also analyzed T cells' activation after inoculation and found that PRV-ΔgE/ΔgI/ΔTK did not activate T cells both recombinant and virulent strains can activate CD4^+^ and CD8^+^ T cells at 72 hpi. Recombinant strains' ability to activate T cells was stronger than that of virulent strain (Fig. 7e, j, o). However, the activation of T cells by PRV-ΔgE/ΔgI/ΔTK was detected in the late stage of infection (200 hpi), mainly manifested as the activation of CD8^+^ T cells (Additional file 5: Figure S5D, S5H and S5L). These data suggest that both PRV-ΔgE/ΔgI/ΔTK and PRV-ΔgE/ΔgI/ΔTK-(CD2v) can stimulate the cellular immune response.

**Fig. 8:**
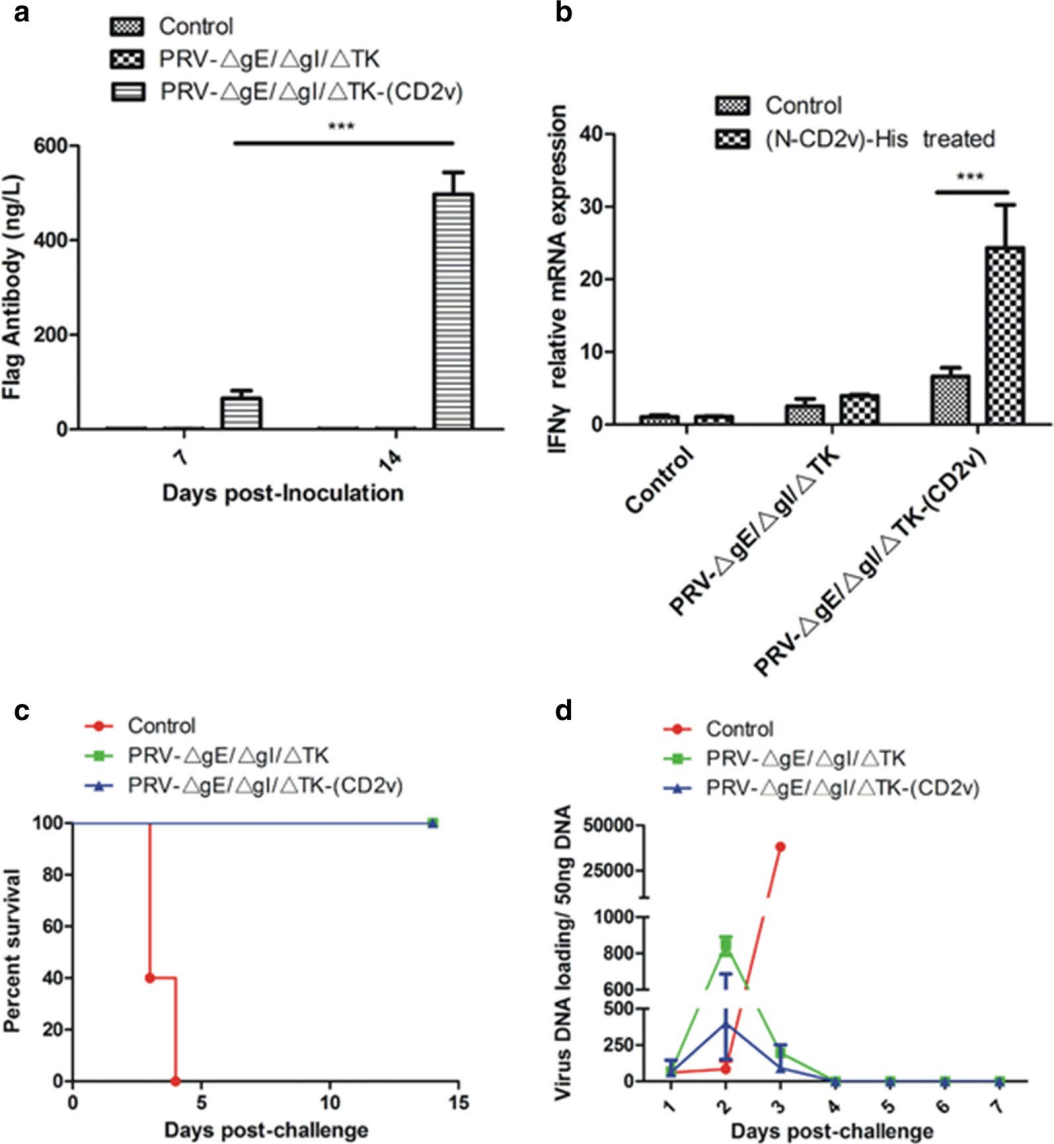
After immunization, the recombinant attenuated virus can produce anti-CD2v specific antibody and specific cell response and has a 100% protective effect against challenge by virulent strains. **a** Anti-CD2v specific antibody can be produced by immunizing the recombinant strain. Five-week-old SPF-ICR mice were immunized with 1 × 10^5^ TCID50 viruses by intramuscular injection in the right hind leg. The second immunization was carried out 7 days after the first immunization. The control group was injected with 100 μL DMEM. About 200 μl blood was collected from the orbit before and 7 days after each immunization for ELSIA to detect Flag antibody (n = 5/each group). **b** IFNγ was detected at high transcription level when PBMC was stimulated with purified (N-CD2v)-His (n = 5/each group). **c** Recombinant strain can 100% protect mice from virulent virus challenge. After the second immunization for 7 days, challenged with 5 × 10^5^ TCID50 PRV-Fa, and the survival curve was drawn (n = 15/each group). **d** There was less detoxification in the recombinant attenuated virus-infected groups in response to the PRV-Fa challenge. After the PRV-Fa challenge, the feces of mice were collected every day to detect the number of virus copies (n = 5/each group). Two-way ANOVA was performed by GraphPad Prism 5.0, GraphPad Software (San Diego, CA, USA), ***p < 0.001

Because we made the fusion expression of CD2v with Flag, the CD2v antibody production was indirectly estimated by anti-Flag reaction using ELISA. The immunization was conducted twice with an interval of 7 days. We only found the Flag antibody in PRV-ΔgE/ΔgI/ΔTK-(CD2v)-infected group (Fig. 8a), and the production of Flag antibody was increased after enhanced immunization. Besides, since IFNγ is a major marker for T cell activation, we also measured the expression of IFNγ induced by CD2v in vitro. On the 7th day after the second immunization, mouse PBMCs were isolated and stimulated with purified (N-CD2v)-His (Additional file 6: Figure S6). We observed a significant increase in the IFNγ mRNA expression in PBMCs (Fig. 8b). These data suggest that the PRV-ΔgE/ΔgI/ΔTK-(CD2v) is highly immunogenic and can induce specific cellular and humoral immune responses in mice.

### Recombinant strains can 100% protect mice from challenge by the virulent strain

To investigate if the recombinant virus could induce protective immunity, the PRV-Fa was used to challenge the mice pretreated with PRV-ΔgE/ΔgI/ΔTK or PRV-ΔgE/ΔgI/ΔTK-(CD2v). As shown in Fig. 8c, the control group died on the 4th day of the PRV-Fa challenge, while both PRV-ΔgE/ΔgI/ΔTK and PRV-ΔgE/ΔgI/ΔTK-(CD2v) immunized groups did not die until the 14th day. Meanwhile, we examined the detoxification effects in mice after the PRV-Fa challenge. The peak of detoxification in the control group lagged behind that in the immunized groups, a large amount of detoxification began on the second day of the PRV-Fa challenge in the immunized groups and the detoxification was not detected until the 4th day, while in the control group, a large amount of detoxification was seen on the 3rd day after the PRV-Fa challenge (Fig. 8d), indicating that the virulent virus could not rapidly replicate in the mice immunized with PRV-ΔgE/ΔgI/ΔTK or PRV-ΔgE/ΔgI/ΔTK-(CD2v). Therefore, immunization with PRV-ΔgE/ΔgI/ΔTK or PRV-ΔgE/ΔgI/ΔTK-(CD2v) can 100% protect the mice challenged by PRV-Fa.

## Discussion

The outbreak of ASF has seriously threatened the development of the pig breeding industry, but there is no effective vaccine to prevent the disease. Various ASFV vaccines have been made, but none has been commercially used [14, 16, 20, 25]. Although some viral vector vaccines have good in vitro proliferation capacity, low production costs, and can effectively activate T cell immune responses [44], many other vaccines have suffered from low yields, high production costs, many side effects, and poor protection capabilities [7, 12, 23]. Pseudorabies virus is a good example of many replication nonessential genes such as gE, gI, TK and so on in its huge genome. While replaced by foreign genes, the virulent virus can become a safe and effective recombinant virus vector vaccine such as JS-2012-ΔgE/gI-E2, PRV SA215/VP2, and PRV-P12A3C [39–41]. The alpha-herpes virus thymidine kinase (TK) gene is unnecessary for virus replication but is a gene related to virulence. It is usually the target gene of choice for constructing live attenuated vaccines with gene deletions and genetically engineered vector vaccines with foreign genes inserted and expressed.

The TK, gE, and gI three-gene deletion strain (PRV TK^−^/gE^−^/gI^−^ (Fa)) has been developed and commercially used as a live attenuated vaccine in China. Compared with current epidemic PRV strains, its safety has been accepted [30]. Currently, the vaccines can target both genotype I and II strains, while type II is the prevalent strain in China. CD2v plays a major role in ASFV immune escape and tissue phagocytosis. As a key virulence gene, CD2v is usually deleted in the development of live attenuated vaccines [45]. Numerous studies have shown that CD2v to provides partial protection as a subunit vaccine, DNA vaccine, and viral vector vaccine, indicating that CD2v can induce immune protection [44]. In this study, we inserted the ASFV EP402R (CD2v) gene into the TK gene site of PRV-ΔgE/ΔgI to generate PRV-ΔgE/ΔgI/ΔTK-(CD2v) by the CRISPR/Cas9 technology [46–48]. This virus strain is quite stable since CD2v is still expressed in infected Vero cells after 20 generations of passages. Previous studies suggest that CD2v processing is mainly depended on the infection of ASFV since the CD2v protein is not processed in uninfected cells [42]. In contrast, we observed several CD2v processed isoforms in pcDNA3.1-EGFP-Flag-CD2v-Flag plasmid transfected and PRV-ΔgE/ΔgI/ΔTK-(CD2v) infected HEK 293 T cells. Thereby, we suggest that the processing of CD2v does not depend on the infection of ASFV, but may be related to cell types and cell death process.

CD2v is related to ASFV tissue phagocytosis [49]. To test whether the insertion of CD2v would change the tissue pathology of PRV-ΔgE/ΔgI/ΔTK, we examined the viral copy number of mouse organ tissues. We found virus nucleic acid only in the various tissues, including the brain of the mice infected with PRV-Fa, but not PRV-ΔgE/ΔgI/ΔTK) or PRV-ΔgE/ΔgI/ΔTK-(CD2v) at 72 hpi. Extending the infection time to 200 h, we detected viral nucleic acids in the brain and lung tissues of mice in the PRV-ΔgE/ΔgI/ΔTK group or PRV-ΔgE/ΔgI/ΔTK-(CD2v) group. But unlike PRV-Bartha, after prolonging the infection time, the viral load in the mouse brain is not higher than that of the virulent strain [32], suggesting that the virulence of the recombinant strain is weaker than PRV-Bartha. Unlike PRV-Fa, the infection with PRV-ΔgE/ΔgI/ΔTK and PRV-ΔgE/ΔgI/ΔTK-(CD2v) in mice did not cause pruritus and death. Systemic inflammation, especially neuroinflammation caused by virulent strains is the major cause of death in mice. IL6 is a major sign of systemic inflammation which can be induced by virulent virus infection such as PRV-Becker infection [32]. But, PRV-ΔgE/ΔgI/ΔTK or PRV-ΔgE/ΔgI/ΔTK-(CD2v) did not cause an IL6 increase in infected mice's tissues and sera. Besides, H&E staining showed that only the PRV-Fa strain could cause brain inflammation in mice. Therefore, the insertion of CD2v does not change the virulence and tissue phagocytosis of the attenuated strain (PRV-ΔgE/ΔgI/ΔTK), and the recombinant strain is safe for mice.

CD2v is a decisive factor for ASFV infection of peripheral blood lymphocytes in *vitro* to inhibit lymphocyte proliferation in response to mitogens [43]. Whether CD2v will inhibit T cell proliferation in *vivo* is still unknown. When we extracted the mouse organs, we found the splenomegaly in the mice inoculated with PRV-ΔgE/ΔgI/ΔTK or PRV-ΔgE/ΔgI/ΔTK-(CD2v), suggesting that the immunization of the attenuated strains activate the mouse immune system leading to the splenomegaly. We found that the spleen congestion filled with a large number of lymphocytes, and the inoculation of these attenuated strains increase CD3^+^, CD3^+^CD4^+^ T cells. However, the ability of PRV-ΔgE/ΔgI/ΔTK-(CD2v) to induce T cell proliferation is weaker than PRV-ΔgE/ΔgI/ΔTK, which is mainly manifested as inhibition of CD8^+^ T cells, suggesting that CD2v in the recombinant strain can still interfere with the proliferation of T cells in vivo, but with less capability. The structure and function of ASFV CD2v protein resemble that of the host CD2 [45]. CD2 can activate T cells after binding to its ligands [50]. Whether CD2v can activate T cells in vivo has not been reported previously. CD69^+^ cells are a major indicator of T cell activation [51]. We analyzed the T cell subtype and found that the recombinant strain can activate T cells after immunization; both CD4^+^ and CD8^+^ T cells are activated, but PRV-ΔgE/ΔgI/ΔTK cannot activate T cells at 72 hpi. PRV-ΔgE/ΔgI/ΔTK can also activate T cells, but this occurs at the late stage (200 hpi) of infection. These data suggest that CD2v can activate T cells in vivo. CD4^+^ delayed-type hypersensitivity-like effector cells are a crucial effector mechanism for protective immunity against PRV [52]. Compared with PRV-ΔgE/ΔgI/ΔTK, the recombinant strain can activate more CD4^+^ T cells. Therefore, the recombinant strain is more suitable for the prevention of pseudorabies.

Adenovirus, vaccinia virus Ankara and alphavirus are often used to develop ASFV virus vector vaccines. They can induce specific antibody production or T cell immune response, but most of their protection capabilities have not been verified [5]. In this study, the production of the specific antibody targeting CD2v has also been manifested by the indirect examination of the anti-Flag antibody production since CD2v is fused with the Flag-tag and the detection of the anti-Flag antibody can represent the expression antigenicity of CD2v protein. But, we can not exclude the other possibilities that may interfere with the CD2v expression or the inability of antibody production associated with CD2v production, processing, or antigen-presenting, so on. It has shown that CD2v protein can protect piglets from the ASFV challenge, even the neutralizing antibody is not present, which implicates the other factors that may be involved in counteracting the viral infection [15]. Previous studies have shown that pigs with depleted CD8^+^ T cells are not immune protected and suggested that a vaccine that can stimulate T-cell-mediated response may also reserve its ability to protect against ASFV infection [19, 53].

Interestingly, our data show that PRV-ΔgE/ΔgI/ΔTK-(CD2v) can effectively activate the immune system to produce specific antibodies with strong immunogenicity. Besides, purified CD2v protein can also induce IFNγ expression, IFNγ is a major marker for T cell activation. Therefore, the PRV-ΔgE/ΔgI/ΔTK-(CD2v) strain can effectively activate specific T cell immune responses.

## Conclusions

In summary, we constructed a PRV attenuated strain expressing ASFV CD2v protein (PRV-ΔgE/ΔgI/ΔTK-(CD2v)) by the CRISPR/Cas9 technology. The recombinant strain showed good safety and immunogenicity in mice. When facing a virulent virus challenge, it has 100% protective ability and can induce CD2v specific humoral and cellular immune responses in mice. Therefore, it may be used as a recombinant vaccine candidate to prevent ASF and Pseudorabies, but further studies are needed to confirm the effectiveness of this attenuated strain in pigs.

## Supplementary information

**Additional file 1**. ** Figure S1**: The infection of a virulent strain caused severe itching in mice. Five-week-old SPF-ICR mice were inoculated with 1 × 10^5^ TCID50 viruses by intramuscular injection in the right hind leg. The control group (Mock) was injected with 100 μL DMEM. The itching symptom appeared in the group inoculated with the virulent strain at about 72 hpi, and the red coil area was the scratched and bitten area of the mouse.

**Additional file 2**. ** Figure S2**: qPCR analyses of viral nucleic acid copies, the detection of viral nucleic acid limit was between 10 and 100 copies. A: pCDH-UL42 plasmid map. B: Standard curve amplification curve, the plasmid of pCDH-UL42 with 1 ng, 0.1 ng, 0.01 ng, 0.001 ng, 0.0001 ng, 0.00001 ng, 0.00001 ng, 0.000001 ng was used for the qPCR reaction. C: Standard curve equation was drawn, the CT value was shown at the horizontal axis, while the vertical axis represented the Log10 plasmid copy number. D: Gel electrophoresis of qPCR products.

**Additional file 3**. **Figure S3**: PRV-ΔgE/ΔgI/ΔTK or PRV-ΔgE/ΔgI/ΔTK-(CD2v) infection showed no difference in spleen weight than the control group (200 hpi). Five-week-old SPF-ICR mice were inoculated with 1 × 10^5^ TCID50 viruses by intramuscular injection in the right hind leg. The control group was injected with 100 μL DMEM. Spleen was collected for weighing at 200 hpi. Unpaired t-test was performed by GraphPad Prism 5.0, GraphPad Software (San Diego, CA, USA), ns (not significant).

**Additional file 4**. ** Figure S4**: PRV-ΔgE/ΔgI/ΔTK and PRV-ΔgE/ΔgI/ΔTK-(CD2v) inoculation led to T cell proliferation at 200 hpi. A-C: The results of CD3^+^ T cell flow analyses. E-G: The results of CD3^+^CD4^+^, CD3^+^CD8^+^ T cell flow analyses. The data showed the changes of the percentage of CD3^+^ (D), CD3^+^CD4^+^ (H), CD3^+^CD8^+^ (I) T cells in total PBMCs. Five-week-old SPF-ICR mice were inoculated with 1 × 10^5^ TCID50 viruses by intramuscular injection in the right hind leg. The control group was injected with 100 μL DMEM. The target cells were collected and analyzed by flow cytometry after 200 h of virus infection. Unpaired t-test was performed by GraphPad Prism 5.0, GraphPad Software (San Diego, CA, USA), *p < 0.05 (n = 5/each group).

**Additional file 5**. **Figure S5**: PRV-ΔgE/ΔgI/ΔTK and PRV-ΔgE/ΔgI/ΔTK-(CD2v) inoculation led to T cells' activation at 200 hpi. A-C: The result of CD3^+^CD69^+^ T cell flow analysis. E-G: The result of CD4^+^CD69^+^ T cell flow analysis. I-K: The result of CD8^+^CD69^+^ T cell flow analysis. The data showed the changes of the percentage of CD3^+^CD69^+^ (D), CD4^+^CD69^+^ (H), CD8^+^CD69^+^ (L) T cells in total PBMCs. Five-week-old SPF-ICR mice were inoculated with 1 × 10^5^ TCID50 viruses by intramuscular injection in the right hind leg. The control group was injected with 100 μL DMEM. The target cells were collected and analyzed by flow cytometry after 200 h of virus infection. Unpaired t-test was performed by GraphPad Prism 5.0, GraphPad Software (San Diego, CA, USA), *p < 0.05, **p < 0.01, ***p < 0.001, (n = 5/each group).

**Additional file 6**. ** Figure S6**: Purification of (N-CD2v)-His recombinant protein. Coomassie brilliant blue staining results, lanes 1 and 7 are markers, lane 2 is the supernatant of EXPi 293 cell lysate transfected with pcDNA3.4 empty vector, lane 3 is EXPi 293 cell transfected with pcDNA3.4-(N-CD2v)-His the supernatant of EXPi 293 cell lysate, lane 4 is the penetrating solution of the transfected pcDNA3.4-(N-CD2v)-His EXPi 293 cell lysate after passing through the nickel column, and lane 5 is the Wash buffer after washing nickel column liquid permeation, lane 6 is the sample eluent collected by the Elution buffer.

## Data Availability

The data used to support the findings of this study are included in this published article.

## Abbreviations

ASF: African swine fever
ASFV: African swine fever virus
PRV: Pseudorabies virus
CPE: Cytopathic effects
DMEM: Dulbecco's modified Eagle's Medium
ELISA: Enzyme-linked immunosorbent assay
CTL: Cytotoxic T lymphocyte
HEK: Human embryonic kidney
FBS: Fetal bovine serum
FITC: Fluorescein isothiocyanate
mAb: Monoclonal antibody
H&E staining: Hematoxylin–Eosin Staining
PBS: Phosphate buffered saline
IgG: Immunoglobulin G
KD: Thousand Daltons
PBMC: Peripheral blood lymphocytes
hpi: Hour post-infection
KO: Knockout
PCR: Polymerase chain reaction
IL6: Interleukin-6
IFN-γ: Interferon-γ
TNF-α: Tumor necrosis factor-α
IL1β: Interleukin1-β
IFNβ: Interferon-β
qPCR: Quantitative polymerase chain reaction
qRT-PCR: Quantitative reverse transcription-polymerase chain reaction
SEM: Standard error of the mean
TCID50: Tissue culture infective dose
MUS: Mouse
F: Forward
R: Reverse
