# Supplementary figures and images for: The recombinant pseudorabies virus expressing African swine fever virus CD2v protein is safe and effective in mice
Source: Virol J. 2020 Nov 16;17:180. doi: 10.1186/s12985-020-01450-7 (PMC7668019; doi:10.1186/s12985-020-01450-7)

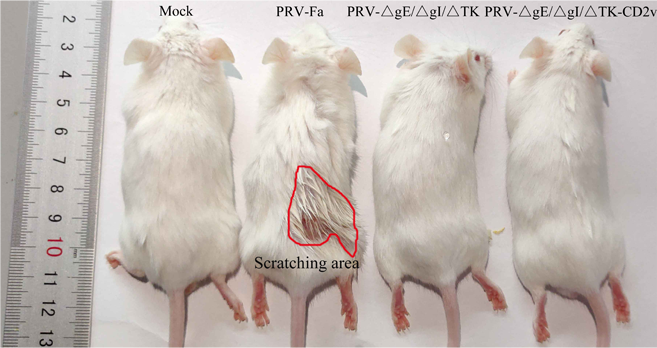

Supplement: Supplementary file 1 — Additional file 1. Figure S1: The infection of a virulent strain caused severe itching in mice. Five-week-old SPF-ICR mice were inoculated with 1 × 10^5^ TCID50 viruses by intramuscular injection in the right hind leg. The control group (Mock) was injected with 100 μL DMEM. The itching symptom appeared in the group inoculated with the virulent strain at about 72 hpi, and the red coil area was the scratched and bitten area of the mouse. [file 12985_2020_1450_MOESM1_ESM.png]

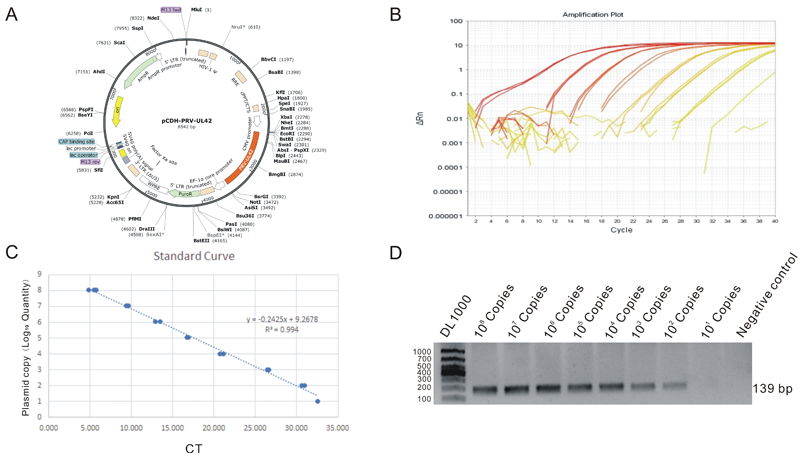

Supplement: Supplementary file 2 — Additional file 2. Figure S2: qPCR analyses of viral nucleic acid copies, the detection of viral nucleic acid limit was between 10 and 100 copies. A: pCDH-UL42 plasmid map. B: Standard curve amplification curve, the plasmid of pCDH-UL42 with 1 ng, 0.1 ng, 0.01 ng, 0.001 ng, 0.0001 ng, 0.00001 ng, 0.00001 ng, 0.000001 ng was used for the qPCR reaction. C: Standard curve equation was drawn, the CT value was shown at the horizontal axis, while the vertical axis represented the Log10 plasmid copy number. D: Gel electrophoresis of qPCR products. [file 12985_2020_1450_MOESM2_ESM.png]

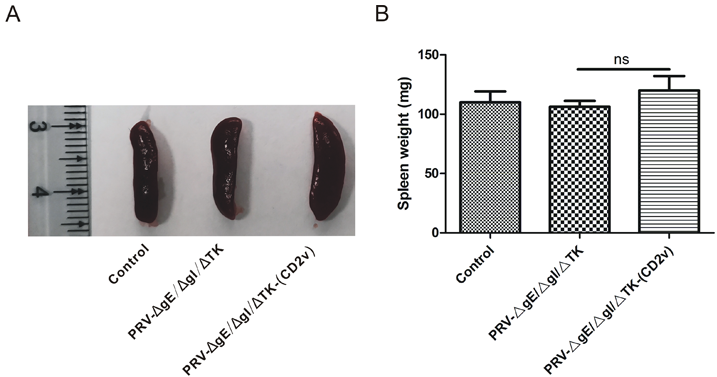

Supplement: Supplementary file 3 — Additional file 3. Figure S3: PRV-ΔgE/ΔgI/ΔTK or PRV-ΔgE/ΔgI/ΔTK-(CD2v) infection showed no difference in spleen weight than the control group (200 hpi). Five-week-old SPF-ICR mice were inoculated with 1 × 10^5^ TCID50 viruses by intramuscular injection in the right hind leg. The control group was injected with 100 μL DMEM. Spleen was collected for weighing at 200 hpi. Unpaired t-test was performed by GraphPad Prism 5.0, GraphPad Software (San Diego, CA, USA), ns (not significant). [file 12985_2020_1450_MOESM3_ESM.png]

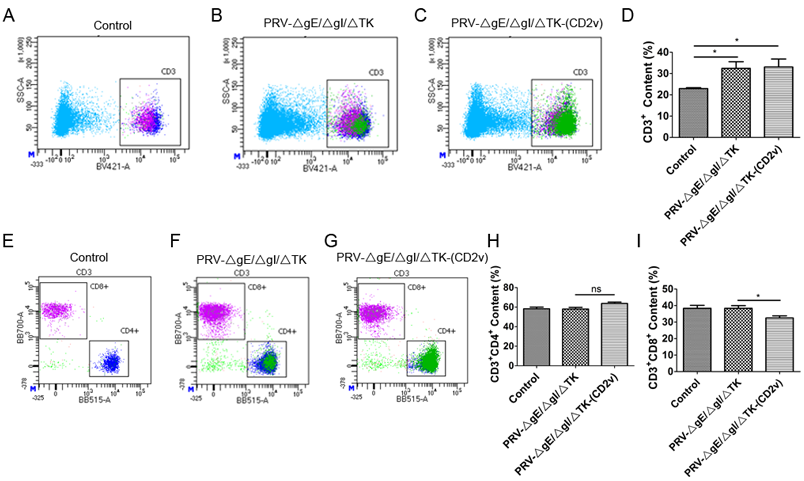

Supplement: Supplementary file 4 — Additional file 4. Figure S4: PRV-ΔgE/ΔgI/ΔTK and PRV-ΔgE/ΔgI/ΔTK-(CD2v) inoculation led to T cell proliferation at 200 hpi. A-C: The results of CD3+ T cell flow analyses. E-G: The results of CD3+CD4+, CD3+CD8+ T cell flow analyses. The data showed the changes of the percentage of CD3+ (D), CD3+CD4+ (H), CD3+CD8+ (I) T cells in total PBMCs. Five-week-old SPF-ICR mice were inoculated with 1 × 10^5^ TCID50 viruses by intramuscular injection in the right hind leg. The control group was injected with 100 μL DMEM. The target cells were collected and analyzed by flow cytometry after 200 h of virus infection. Unpaired t-test was performed by GraphPad Prism 5.0, GraphPad Software (San Diego, CA, USA), *p < 0.05 (n = 5/each group). [file 12985_2020_1450_MOESM4_ESM.png]

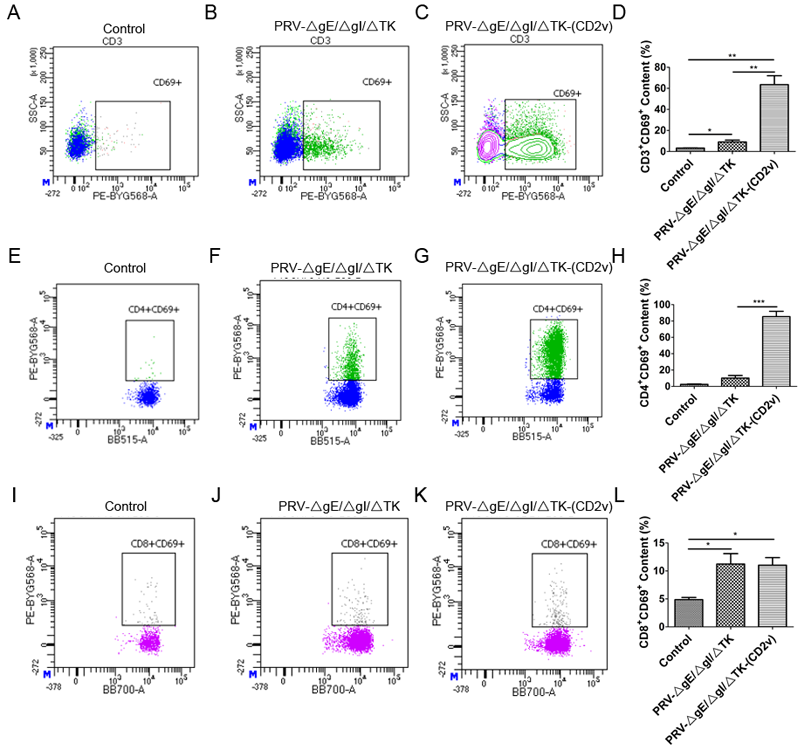

Supplement: Supplementary file 5 — Additional file 5. Figure S5: PRV-ΔgE/ΔgI/ΔTK and PRV-ΔgE/ΔgI/ΔTK-(CD2v) inoculation led to T cells' activation at 200 hpi. A-C: The result of CD3+CD69+ T cell flow analysis. E-G: The result of CD4+CD69+ T cell flow analysis. I-K: The result of CD8+CD69+ T cell flow analysis. The data showed the changes of the percentage of CD3+CD69+ (D), CD4+CD69+ (H), CD8+CD69+ (L) T cells in total PBMCs. Five-week-old SPF-ICR mice were inoculated with 1 × 10^5^ TCID50 viruses by intramuscular injection in the right hind leg. The control group was injected with 100 μL DMEM. The target cells were collected and analyzed by flow cytometry after 200 h of virus infection. Unpaired t-test was performed by GraphPad Prism 5.0, GraphPad Software (San Diego, CA, USA), *p < 0.05, **p < 0.01, ***p < 0.001, (n = 5/each group). [file 12985_2020_1450_MOESM5_ESM.png]

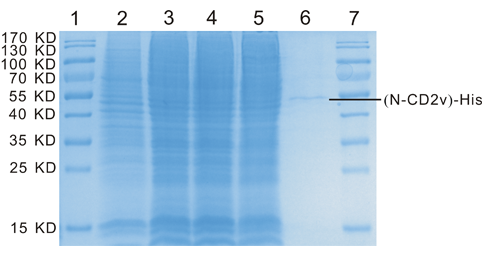

Supplement: Supplementary file 6 — Additional file 6. Figure S6: Purification of (N-CD2v)-His recombinant protein. Coomassie brilliant blue staining results, lanes 1 and 7 are markers, lane 2 is the supernatant of EXPi 293 cell lysate transfected with pcDNA3.4 empty vector, lane 3 is EXPi 293 cell transfected with pcDNA3.4-(N-CD2v)-His the supernatant of EXPi 293 cell lysate, lane 4 is the penetrating solution of the transfected pcDNA3.4-(N-CD2v)-His EXPi 293 cell lysate after passing through the nickel column, and lane 5 is the Wash buffer after washing nickel column liquid permeation, lane 6 is the sample eluent collected by the Elution buffer. [file 12985_2020_1450_MOESM6_ESM.png]
